# Complete genome sequence of *Vibrio cholerae* LK-18 isolated from tail-rotted *Procambarus clarkii*

**DOI:** 10.1128/mra.00471-24

**Published:** 2024-08-29

**Authors:** Mingshu Yang, Jinxian Lv, Kai Li, Haojie Lu, Yongjie Wang

**Affiliations:** 1 College of Food Science and Engineering, Hainan Tropical Ocean University; Marine Food Engineering Technology Research Center of Hainan Province; Collaborative Innovation Center of Marine Food Deep Processing, Sanya, China; 2 College of Food Science and Technology, Shanghai Ocean University, Shanghai, China; University of Maryland School of Medicine, Baltimore, Maryland, USA

**Keywords:** *Vibrio cholerae*, *Procambarus clarkii*, tail-rotted disease, genome sequence

## Abstract

Isolated from tail-rotted *Procambarus clarkii*, the pathogenic bacterium *Vibrio cholerae* LK-18 features two circular chromosomes: chromosome I (2,895,335 bp) and chromosome II (1,175,190 bp). The genome includes 3,522 open reading frames, 100 tRNA genes, and 31 rRNA genes, and it harbors the *Vibrio cholera* cytolysin and chitinase genes.

## ANNOUNCEMENT

In May 2018, a batch of tail-rotted *Procambarus clarkii* was collected from Mianchang, Pudong New Area, Shanghai. Using thiosulfate citrate bile-salts sucrose (TCBS) agar for cultivation and 16S rRNA sequencing for identification, we isolated a potentially pathogenic bacterium from the tail tissue, identified as *Vibrio cholerae* LK-18 (1). To gain an understanding of the mechanisms and pathogenesis of *V. cholerae* infections in crayfish at the genomic level, we conducted whole-genome sequencing and analysis of the isolate.

The LK-18 was inoculated into 200 mL of tryptone soya broth (TSB) medium and incubated with shaking at 200 rpm for 8 hours at 30°C. Genomic DNA was extracted using Wizard Genomic DNA Purification Kit (Promega, USA) and sequenced using a combination of PacBio Sequel IIe and Illumina sequencing platforms. The DNA samples were sheared into 400–500 bp fragments using a Covaris M220 Focused Acoustic Shearer. Illumina sequencing libraries were prepared using the Nextflex Rapid DNA-Seq Kit (Bioo Scientific, Austin, TX, USA). The libraries were used for paired-end Illumina sequencing (2 × 150 bp) on Illumina Novaseq 6000 (Illumina Inc., San Diego, CA, USA). The raw Illumina sequencing reads generated from the paired-end library were subjected to quality filtered using fastp v0.23.0. A total of 4,946,769 paired-end reads were generated, yielding a sequencing depth of over 300×. For PacBio sequencing, the high-quality genomic DNA (OD260/280 = 1.8–2.0, total amount ≥10 µg) was fragmented to ~10 kb (SMRTbell Prep Kit 3.0, Pacific Biosciences, CA, USA). The PacBio library was prepared and sequenced on a single-molecule real-time (SMRT) cell, generating a total of 177,156 reads, with an average length of 6,749bp, a longest read of 133,496bp, and an *N*_50_ value of 11,161bp. The clean short reads and HiFi reads were assembled to construct complete genomes using Unicycler v0.4.8 (2) and uses Pilon v1.22 to polish the assembly using short-read alignments. The coding sequences of chromosome and plasmid were predicted using Glimmer v3.02 (3) and GeneMarkS v4.25 (4), respectively. tRNA-scan-SE v2.0 (5) was used for tRNA prediction, Barrnap v0.9 was used for rRNA prediction, and PGAP v3.0 was performed for annotation (6).

**Fig 1:**
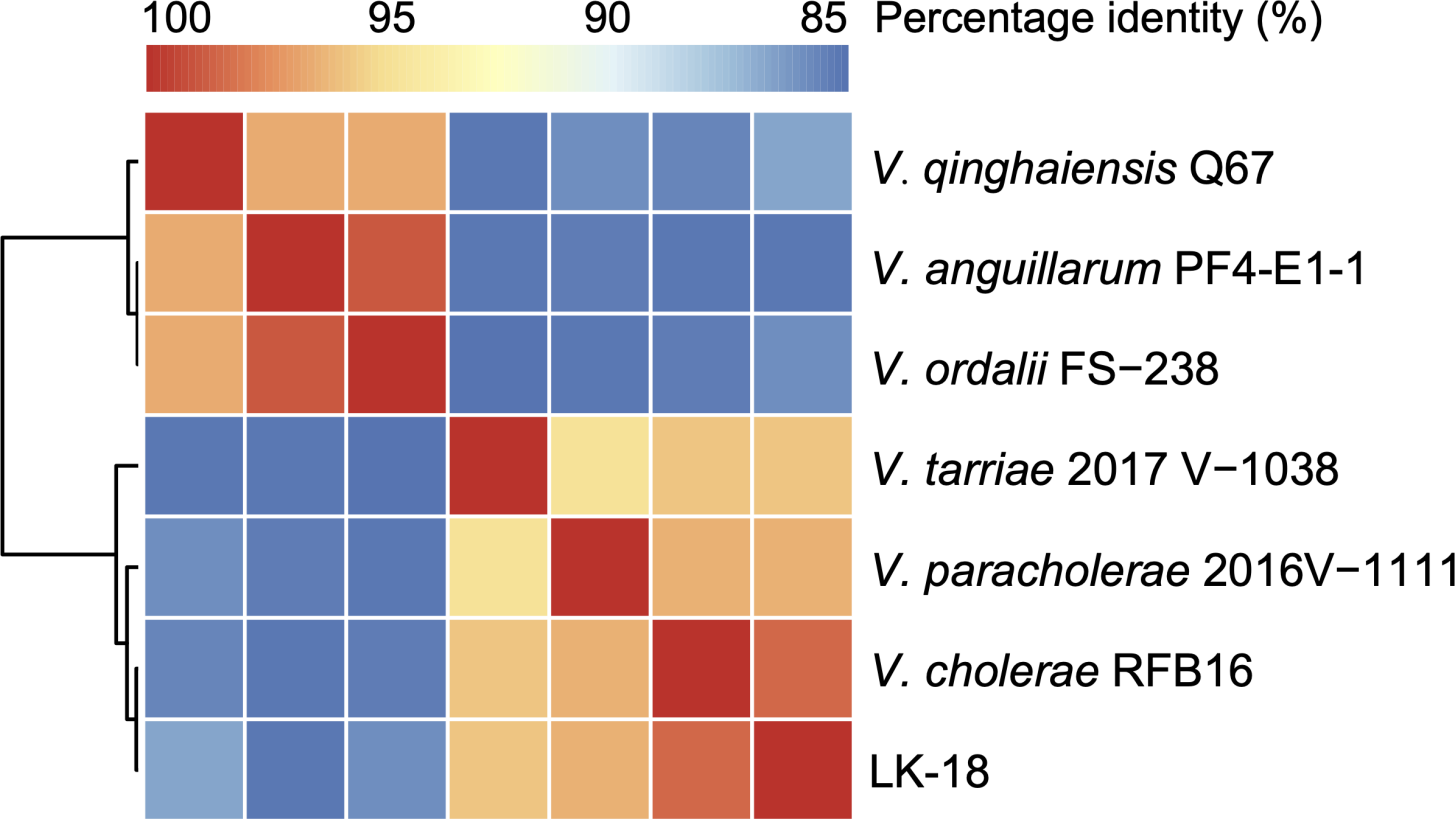
Heatmap of the ANI (%) of *V. cholerae* LK-18. *V. cholerae* LK-18 versus the other six *Vibrio* strains of *V. qinghaiensis* Q67 (GCF_002257545.1), *V. anguillarum* PF4-E1-1 (GCF_003390515.1), *V. ordalii* FS-238 (GCF_000287155.2), *V. tarriae* 2017 V-1038 (GCF_003311805.1), *V. paracholerae* 2016V-1111 (GCF_003311965.1), and *V. cholerae* RFB16 (GCF_008369605.1).

The LK-18 contains chromosome I with a length of 2,895,335 bp and chromosome II with a length of 1,175,190 bp. The guanine and cytosine (GC) contents were 47.94% and 46.88% for chromosomes I and II, respectively. The genome encodes a total of 3,522 open reading frames (ORFs), including 100 tRNA and 31 rRNA. Chromosome I contains 2,490 ORFs, 31 rRNA, and 96 tRNA. Chromosome II contains 1,032 ORFs and 4 tRNA. A whole-genome average nucleotide identity (ANI) analysis was conducted on LK-18 and six known *Vibrio* strains using pyani (7). The analysis revealed an ANI value of 98.2% between LK-18 and *V. cholerae* RFB16, indicating a close genetic relationship and supporting the classification of LK-18 within the *V. cholerae* species (Fig. 1).

In the context of tail rot disease, the LK-18 lacks the *ctx* and CFA genes (8, 9). However, the presence of the chitinase and *Vibrio cholera* cytolysin virulence gene indicates the potential for the expression of chitinase and hemolysin, which could disrupt the host’s shell and lead to cell damage or death (10).

## Data Availability

This Whole Genome project has been deposited in GenBank under the locus tag prefix VFH86. The genome accession number is GCF_039602145.1. The raw sequencing reads were deposited to the GenBank under the accession numbers SRR28906461 and SRR28906462.
